# Knockout of *mlaA* increases *Escherichia coli* virulence in a silkworm infection model

**DOI:** 10.1371/journal.pone.0270166

**Published:** 2022-07-13

**Authors:** Haruka Nasu, Riko Shirakawa, Kazuyuki Furuta, Chikara Kaito

**Affiliations:** Graduate School of Medicine, Dentistry, and Pharmaceutical Sciences, Okayama University, Kita-ku, Okayama, Japan; CINVESTAV-IPN, MEXICO

## Abstract

The *mlaA* gene encodes a lipoprotein to maintain an outer membrane lipid asymmetry in gram-negative bacteria. Although the role of *mlaA* in bacterial virulence has been studied in several bacterial species, there are no reports of its role in *E*. *coli* virulence. In this study, we found that knockout of *mlaA* in *E*. *coli* increased its virulence against silkworms. The *mlaA-*knockout mutant was sensitive to several antibiotics and detergents, but resistant to vancomycin and chlorhexidine. The *mlaA*-knockout mutant grew faster than the parent strain in the presence of silkworm hemolymph. The *mlaA*-knockout mutant also produced a larger amount of outer membrane vesicles than the parent strain. These findings suggest that *mlaA* knockout causes *E*. *coli* resistance to specific antimicrobial substances and increases outer membrane vesicle production, thereby enhancing *E*. *coli* virulence properties in the silkworm infection model.

## Introduction

The outer membrane of gram-negative bacteria, including *E*. *coli*, has a lipid asymmetry, in which the outer leaflet comprises lipopolysaccharide (LPS) and the inner leaflet comprises phospholipids [1]. The outer leaflet has low fluidity and contributes to inhibit the invasion of various antibiotics and foreign chemicals into bacterial cells [2]. The outer membrane lipid asymmetry is maintained by the Mla system, phospholipase A (PldA), and LPS palmitoyltransferase (PagP) [3]. PldA and PagP contribute to maintain the lipid asymmetry by degrading phospholipids in the outer leaflet of the outer membrane. The Mla system consists of 6 proteins, MlaA, MlaB, MlaC, MlaD, MlaE, and MlaF, that transport phospholipids from the outer leaflet of the outer membrane into the inner membrane. MlaA is a lipoprotein locating at the outer membrane that transfers phospholipids to MlaC, a periplasmic protein [4]. MlaC transfers phospholipids to the MlaFEDB complex in the inner membrane, and MlaFEDB then inserts the phospholipids into the inner membrane.

Knockout of the Mla system destroys the lipid asymmetry of the outer membrane and increases the sensitivity to various antimicrobial substances in many gram-negative bacteria. Knockout of MlaA causes bacterial sensitivity to various antimicrobial substances; sodium dodecyl sulfate (SDS)/EDTA in *E*. *coli* [4,5], hydrophobic antibiotics such as erythromycin, rifampicin, azithromycin, and polymyxin E in *Haemophilus influenzae* [6]; polymyxin B and vancomycin in *Neisseria gonorrhoeae* [7]; and tetracycline, ciprofloxacin, chloramphenicol, cathelicidin, and LL-37 in *P*. *aeruginosa* [8,9]. Knockout of MlaC also causes sensitivity to macrolides and fluoroquinolones in *Burkholderia* species [10], and gentamycin, novobiocin, and rifampicin in *Acinetobacter baumanii* [11]. In contrast, however, the MlaA-knockout mutant of *E*. *coli* shows resistance to chlorhexidine [12]. *E*. *coli* mutants resistant to the antimicrobial peptide aenicin-3 carry mutations in the *mlaA* gene or the *mlaBCDEF* operon [13]. The characteristics of antibiotics for which knockout of the Mla system causes bacterial sensitivity or resistance, however, are unknown.

Knockout of MlaA exerts both negative and positive effects on the virulence properties of various bacterial species. In *Shigella flexneri*, knockout of MlaA decreases bacterial spreading among host cells [14,15]. In *H*. *influenzae* and *H*. *parasuis*, knockout of MlaA attenuates bacterial infectivity in host epithelial cells and decreases the bacterial burden in a mouse model [6,16]. In *Burkholderia pseudomallei*, knockout of MlaA decreases bacterial persistence in mouse spleen [17]. On the other hand, in *N*. *gonorrhoeae* and *Vibrio cholerae*, knockout of MlaA increases the bacterial number in the mouse genital tract or gut [7,18]. In *H*. *influenzae*, *A*. *baumannii*, *N*. *gonorrhoeae*, and *V*. *cholerae*, knockout of MlaA increases the production of outer membrane vesicles (OMV), which have various functions in infectious processes, such as absorbing antimicrobial molecules and transporting toxins to host cells [7,18–21]. In *P*. *aeruginosa*, knockout of MlaA decreases bacterial virulence in a mouse lung infection model [9], but increases bacterial virulence in a fruit fly infection model [8]. How MlaA affects *E*. *coli* virulence, however, is unclear.

We previously investigated gene mutations that upregulate *E*. *coli* virulence properties using a silkworm infection model [22,23]. In the present study, we searched *E*. *coli* mutants with high virulence from among *E*. *coli* mutants resistant to vancomycin and found that knockout of MlaA upregulates *E*. *coli* virulence properties.

## Results

### Knockout of *mlaA* increases *E*. *coli* killing activity against silkworms

**Fig 1:**
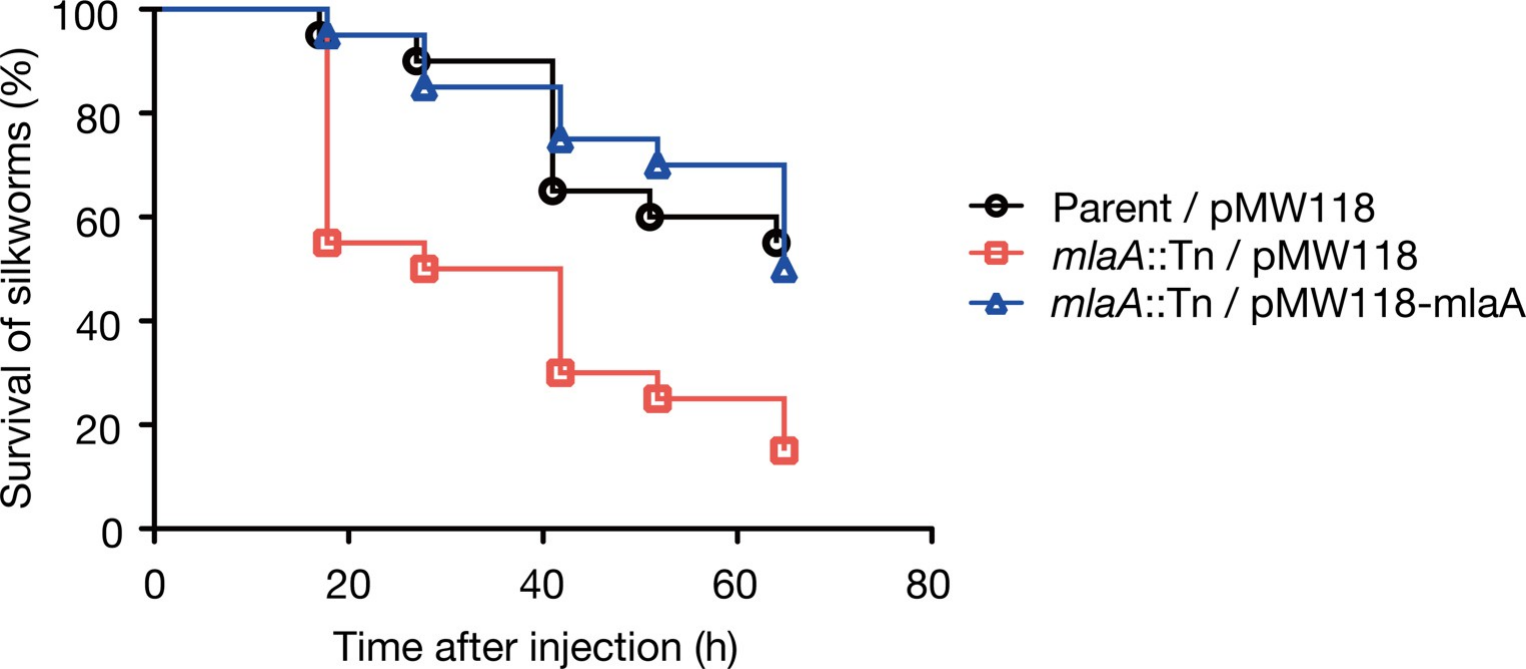
Knockout of *mlaA* increases *E*. *coli* virulence against silkworms. Silkworm killing activity of the parent strain transformed with an empty vector (Parent/pMW118), the *mlaA-*knockout mutant transformed with an empty vector (*mlaA*::Tn/pMW118), and the *mlaA-*knockout mutant transformed with pMW118-mlaA (*mlaA*::Tn/pMW118-mlaA) was examined. Silkworms were injected with bacterial cells (2 x 10^8^ CFU) and survival was monitored. The experiment was performed twice and the data were pooled (n = 20). Log-rank test p value was less than 0.05 between Parent/pMW118 and *mlaA*::Tn/pMW118, or between *mlaA*::Tn/pMW118 and *mlaA*::Tn/pMW118-mlaA.

**Fig 2:**
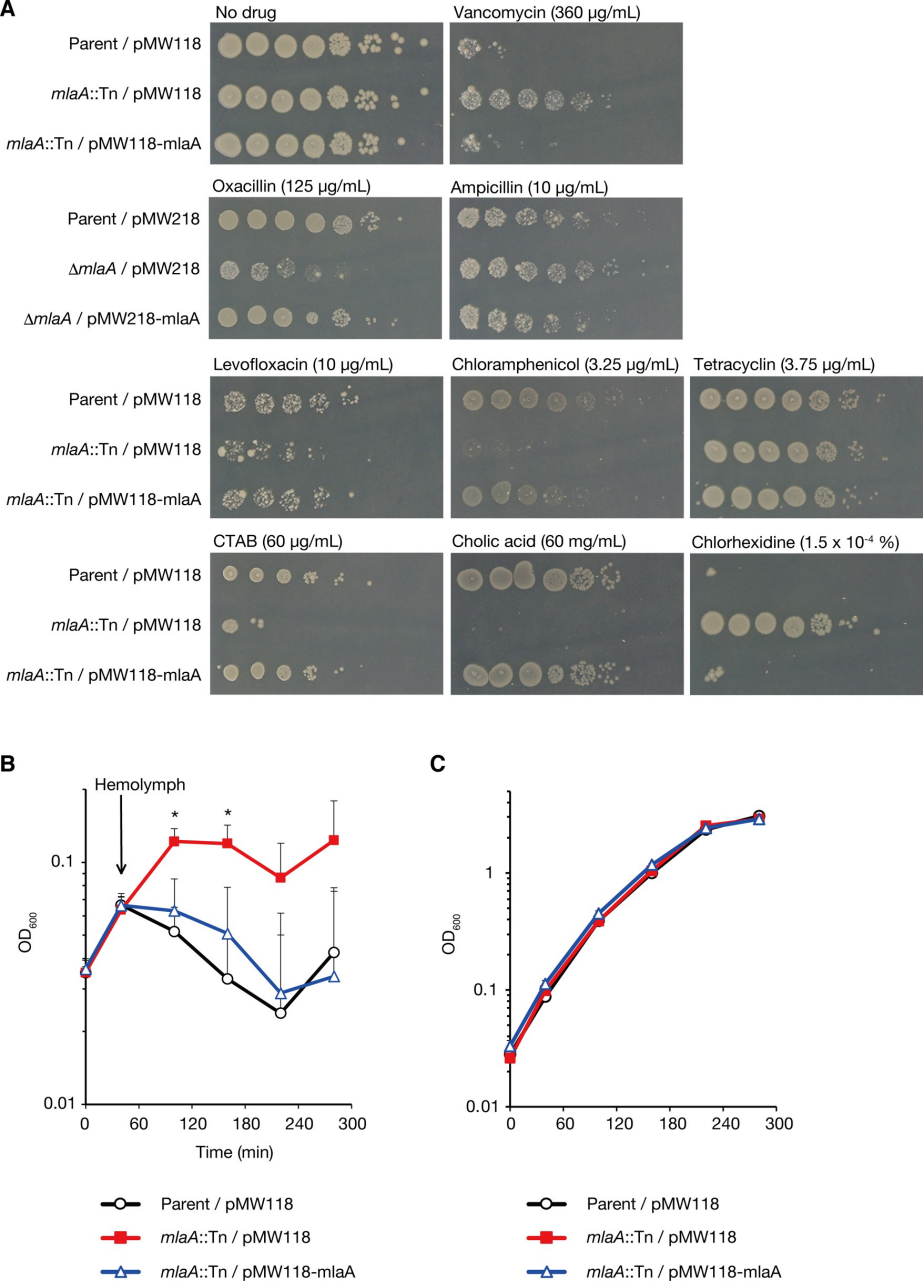
The *mlaA-*knockout mutant exhibits various sensitivities to antibiotics and resistance to silkworm antimicrobial substances. (A) *E*. *coli* overnight culture of the Parent/pMW118, *mlaA*::Tn/pMW118, or *mlaA*::Tn/pMW118-mlaA strain was 10-fold serially diluted; spotted onto LB agar plates supplemented with or without vancomycin, levofloxacin, chloramphenicol, tetracycline, CTAB, cholic acid, or chlorhexidine; and incubated at 37˚C. To examine the sensitivity to oxacillin and ampicillin, the *mlaA* deletion mutant (markerless deletion mutant of *mlaA*) transformed with pMW218 (Δ*mlaA*/pMW218) and the *mlaA* deletion mutant transformed with pMW218-mlaA (Δ*mlaA*/pMW218-mlaA) were used. (B) *E*. *coli* strains of the Parent/pMW118, *mlaA*::Tn/pMW118, or *mlaA*::Tn/pMW118-mlaA were aerobically cultured in LB medium and silkworm hemolymph was added to the bacterial culture at 40 min after the bacterial inoculation. The vertical axis represents the OD_600_ value of the bacterial culture, and the horizontal axis represents the culture time. The means ± standard errors from 5 independent experiments are shown. Star indicates Student t-test p value less than 0.05 between the Parent/pMW118 vs. *mlaA*::Tn/pMW118, and between *mlaA*::Tn/pMW118 vs. *mlaA*::Tn/pMW118-mlaA. (C) *E*. *coli* strains of the Parent/pMW118, *mlaA*::Tn/pMW118, or *mlaA*::Tn/pMW118-mlaA were aerobically cultured in LB medium and the OD_600_ values of the cultures were measured.

We previously revealed that amino acid substitutions in LptD or LptE, the LPS transporter subunits, as well as knockout of OpgG or OpgH, the synthetases for osmoregulated periplasmic glucan, cause *E*. *coli* resistance against vancomycin and increase *E*. *coli* killing activity against silkworms [22,23]. Based on this finding, we hypothesized the existence of *E*. *coli* genes whose knockout could lead to high vancomycin resistance as well as high killing activity against silkworms. By searching *E*. *coli* gene knockout mutants showing vancomycin resistance from a transposon mutant library, we identified 50 gene knockout mutants exhibiting vancomycin resistance (**Table 1**). We evaluated the killing activity of the vancomycin-resistant mutants against silkworms, and found that the *mlaA*-knockout mutant killed silkworms faster than the parent strain (**Table 1**, **Fig 1**). The increased killing activity of the *mlaA*-knockout mutant was blocked by introducing the intact *mlaA* gene (**Fig 1**). In addition, the *mlaA*-knockout mutant exhibited better growth than the parent strain in the presence of vancomycin, and the vancomycin resistance was decreased to the parent level by introducing the intact *mlaA* gene (**Fig 2A**). These results suggest that *mlaA* knockout leads to vancomycin resistance and increases *E*. *coli* virulence against silkworms.

**Table 1:** Silkworm-killing activities of transposon mutants exhibiting resistance against vancomycin.

Strain ID	Gene	Product	Survival (%)
JD20172	*insH1*	Transposase InsH for insertion seguence element IS5A	100
JD20241	*carB*	Carbamoyl-phosphate synthase large chain	60
JD20379	*acnB*	Aconitate hydratase B	20
JD20391	*hpt*	Hypoxanthine phosphoribosyltransferase	40
JD20727	*ybbO*	Uncharacterized oxidoreductase	20
JD20913	*segA*	Endonuclease segA	80
JD21066	*galU*	UTP-glucose-1-phosphate uridylyltransferase	100
JD21400	*psuT*	Putative pseudouridine transporter	60
JD21547	*crr*	PTS system glucose-specific EIIA component	40
JD21607	*guaB*	Inosine-5’-monophosphate dehydrogenase	100
JD21620	*ndk*	Nucleoside diphosphate kinase	80
JD21662	*mltA*	Membrane-bound lytic murein transglycosylase A	60
JD21673	*nlpI*	Lipoprotein NlpI	60
JD21732	*yibB*	Protein HtrL	100
JD21864	*ybiS*	Probable L, D-transpeptidase YbiS	100
JD22094	*opgG*	Glucans biosynthesis protein G	ND
JD22095	*opgH*	Glucans biosynthesis glucosyltransferase H	ND
JD22143	*yceG*	Endolytic murein transglycosylase	80
JD22152	*ycfL*	Uncharacterized protein YcfL	100
JD22156	*lpoB*	Penicillin-binding protein activator LpoB	80
JD23323	*mlaA*	Intermembrane phospholipid transport system lipoprotein MlaA	0
JD23420	*uraA*	Uracil permease	100
JD23606	*proX*	Glycine betaine/proline betain-binding periplasmic protein	100
JD23607	*ygaZ*	Inner membrane protein YgaZ	100
JD23873	*gshB*	Glutathione synthetase	100
JD23934	*yghQ*	Inner membrane protein YghQ	100
JD23935	*yghR*	Uncharacterized ATP-binding protein YghR	80
JD23938	*yghS*	Uncharacterized ATP-binding protein YghS	100
JD23939	*yghT*	Uncharacterized ATP-binding protein YghT	100
JD24024	*tolC*	Outer membrane protein TolC	100
JD24230	*mlaC*	Intermembrane phospholipid transport system binding protein MlaC	60
JD24231	*mlaD*	Intermembrane phospholipid transport system binding protein MlaD	40
JD24242	*yhbJ*	Rnase adapter protein RapZ	80
JD24280	*sspA*	Glutamyl endopeptidase	100
JD24335	*dusB*	tRNA-dihydrouridine syntaseB	100
JD24462	*waaY*	Lipopolysaccharide core heptose(Ⅱ) kinase RfaY	80
JD24466	*waaR*	Lipopolysaccharide 1,2-glucosyltransferase	100
JD24468	*waaB*	Lipopolysaccharide 1,6-galactosyltransferase	100
JD24476	*waaQ*	Lipopolysaccharide core heptosyltransferase RfaQ	100
JD24492	*yicC*	UPF0701 protein YicC	100
JD24700	*wecA*	Undecaprenyl-phosphate alpha-N-acetylglucosaminly 1-phosphate transferase	60
JD25777	*slt*	Soluble lytic murein transglycosylase	20
JD26183	*bioB*	Biotin synthase	80
JD26764	*glf*	UDP-galactopyranose mutase	60
JD27118	*mltB*	Membrane-bound lytic murein transglycosylase B	100
JD27649	*hycB*	Formate hydrogenlyase subunit 2	100
JD27708	*mlaE*	Intermembrane phospholipid transport system permease protein MlaE	60
JD27710	*mlaF*	Intermembrane phospholipid transport system ATP-binding protein MlaF	80
JD27958	*citC*	[Citrate[pro-3S]-lyase]ligase	80

Transposon mutants exhibiting resistance to vancomycin are listed. Bacterial solutions (2 x 10^8^ CFU) were injected into silkworms (n = 5) and percent survival at 2 days post infection was measured and is presented as “Survival”. Percent survival of the parent strain was 100%. ND, not determined.

### Knockout of *mlaA* alters *E*. *coli* sensitivity to various antimicrobial molecules

Next, we examined whether *mlaA* knockout alters *E*. *coli* sensitivity to various antimicrobial molecules. The *mlaA*-knockout mutant showed less growth than the parent strain in the presence of antibiotics such as levofloxacin, chloramphenicol, and oxacillin, and detergents such as cetyltrimethylammonium bromide (CTAB) and cholic acid (**Fig 2A**). The growth of the *mlaA*-knockout mutant was indistinguishable from that of the parent strain in the presence of tetracycline (**Fig 2A**). Consistent with a previous report [12], the *mlaA*-knockout mutant showed better growth than the parent strain in the presence of chlorhexidine (**Fig 2A**). The alteration of drug sensitivity in the *mlaA*-knockout mutant was restored to the parent strain level by introducing the intact *mlaA* gene (**Fig 2A**). These results suggest that *mlaA* knockout alters *E*. *coli* sensitivity to various antimicrobial molecules.

To clarify the molecular mechanisms by which *mlaA* knockout increases *E*. *coli* virulence against silkworms, we examined bacterial growth in the presence of silkworm hemolymph in which antimicrobial peptides were induced by injection of heat-killed bacteria [24]. The *mlaA*-knockout mutant grew faster than the parent strain in the presence of silkworm hemolymph (**Fig 2B**). The faster growth of the *mlaA*-knockout mutant in the presence of silkworm hemolymph was abolished by introducing the intact *mlaA* gene (**Fig 2B**). In contrast, in the absence of silkworm hemolymph, the growth of the *mlaA*-knockout mutant was indistinguishable from that of the parent strain (**Fig 2C**). These results suggest that the *mlaA*-knockout confers *E*. *coli* resistance to silkworm immune mechanisms.

### Knockout of *mlaA* increases OMV production

**Fig 3:**
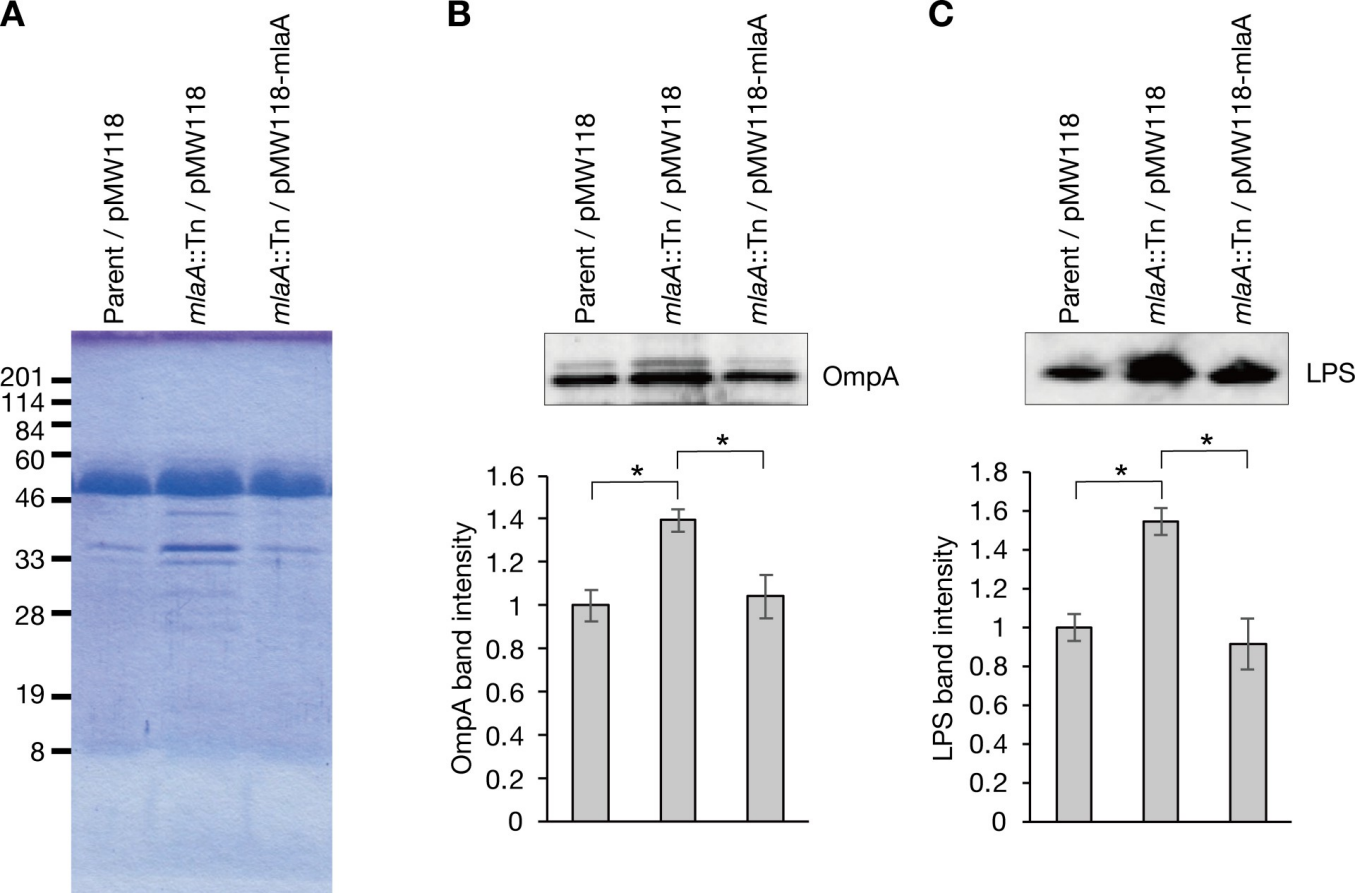
Knockout of *mlaA* increases OMV production. (A) Culture supernatants of the Parent/pMW118, *mlaA*::Tn/pMW118, or *mlaA*::Tn/pMW118-mlaA strains were ultracentrifuged and the precipitates were electrophoresed in a SDS polyacrylamide gel. The gel was stained by Coomassie brilliant blue. (B, C) The OMV fraction obtained in (A) was subjected to Western blot analysis using an anti-OmpA antibody (B) or an anti-LPS antibody (C). Lower graph indicate the relative band intensity compared with that in the parent strain. Data shown are means ± standard errors from three independent experiments. The asterisk represents a p value less than 0.05 (Student’s *t* test).

Knockout of *mlaA* leads to increased production of OMVs in *N*. *gonorrhoeae*, *H*. *influenza*, *V*. *cholerae* [7,20]. We examined whether *mlaA* knockout increases OMV production in *E*. *coli*. SDS-polyacrylamide gel electrophoresis analysis revealed that the amounts of proteins with about 33 kDa increased in the OMV fraction of the *mlaA*-knockout mutant compared with the parent strain (**Fig 3A, S1 Raw images**). Western blot analysis revealed that the amounts of OmpA and LPS, which are components of OMV, increased in the OMV fraction of the *mlaA*-knockout mutant compared with the parent strain (**Fig 3B and 3C, S1 Raw images**). The increase in OmpA and LPS was decreased by introducing the intact *mlaA* gene (**Fig 3B and 3C, S1 Raw images**). These results suggest that *mlaA* knockout increases OMV production.

### Knockout of *pldA* does not affect *E*. *coli* killing activity against silkworms

**Fig 4:**
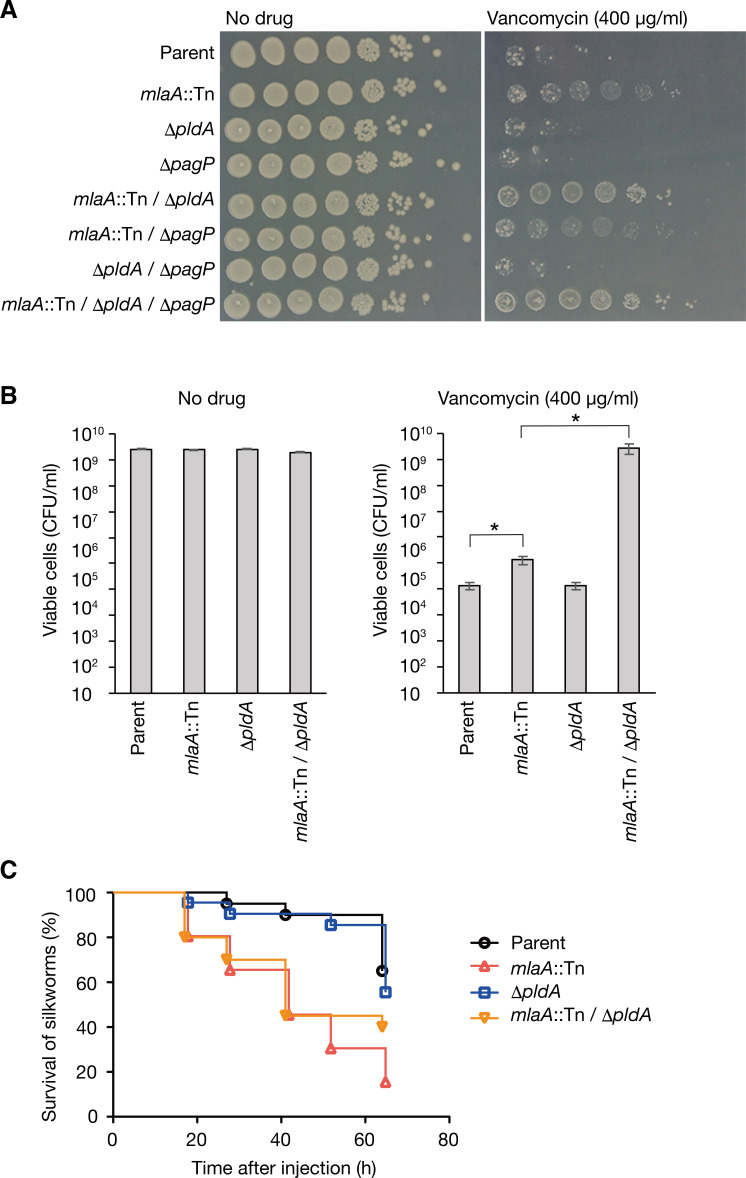
Knockout of *pldA* does not affect silkworm-killing activity. (A) *E*. *coli* overnight culture of the Parent, *mlaA*::Tn, Δ*pldA*, Δ*pagP*, *mlaA*::Tn/Δ*pldA*, *mlaA*::Tn/Δ*pagP*, Δ*pldA*/Δ*pagP*, or *mlaA*::Tn/Δ*pldA*/Δ*pagP* strain was 10-fold serially diluted, spotted onto LB agar plates supplemented with or without vancomycin (400 μg/ml), and incubated at 37˚C. (B) *E*. *coli* overnight culture of the Parent, *mlaA*::Tn, Δ*pldA*, or *mlaA*::Tn/Δ*pldA* strain was 10-fold serially diluted, spread onto LB agar plates supplemented with or without vancomycin (400 μg/ml), and incubated at 37˚C. The appeared colonies were counted and CFU/ml was calculated. Data shown are means ± standard errors from three independent experiments. The asterisk represents a p value less than 0.05 (Student’s *t* test). (C) Silkworms were injected with bacterial cells (2 x 10^8^ CFU) of the Parent, *mlaA*::Tn, Δ*pldA*, or *mlaA*::Tn/Δ*pldA*, and survival was monitored. The experiment was performed twice and the data were pooled (n = 20). Log-rank test p value was less than 0.05 between Parent and *mlaA*::Tn, or between Δ*pldA* and *mlaA*::Tn/Δ*pldA*.

PldA and PagP degrade phospholipids of the outer membrane and contribute to maintain the lipid asymmetry of the outer membrane independently of the Mla system [3]. Double knockout of *mlaA* and *pldA* leads to a higher accumulation of phospholipids in the outer membrane than the respective single knockouts [25]. We examined whether double knockout of *mlaA* and *pldA*, or *mlaA* and *pagP* increases vancomycin resistance. The growth of single knockout mutants of *pldA* or *pagP* was indistinguishable from that of the parent strain in the presence of vancomycin (**Fig 4A**). Double knockout mutants of *mlaA* and *pldA* showed slightly better growth than the respective single knockout mutants in the presence of vancomycin (**Fig 4A**). Colony forming unit assay confirmed the different sensitivity to vancomycin between the *mlaA*-knockout mutant and the *mlaA/pldA* double knockout mutant (**Fig 4B**). The growth of double knockout mutants of *mlaA* and *pagP* was similar to that of the *mlaA*-knockout mutant in the presence of vancomycin (**Fig 4A**). These results suggest that knockouts of *mlaA* and *pldA* additively increase vancomycin resistance.

Based on these observations, we examined the effect of *pldA* knockout on *E*. *coli* virulence against silkworms. The *pldA* single knockout mutant killed silkworms with a similar time course as the parent strain (**Fig 4C**). The *mlaA/pldA* double knockout mutant killed silkworms with a similar time course as the *mlaA* single knockout mutant (**Fig 4C**). Thus, the *pldA* knockout does not increase silkworm killing activity.

## Discussion

The findings of the present study revealed that knockout of *mlaA*, which maintains the lipid asymmetry of the outer membrane leads to increased virulence of *E*. *coli* against silkworms. The *mlaA*-knockout mutant exhibited increased OMV production, resistance to vancomycin, and increased killing activity against silkworms. Thus, this study unveiled a novel function of *mlaA* in *E*. *coli* virulence properties.

Knockout of *mlaA* increases bacterial virulence not only in *E*. *coli*, but also in *N*. *gonorrhoeae*, *V*. *cholerae*, and *P*. *aeruginosa* [7,8,18]. In contrast, knockout of *mlaA* attenuates bacterial virulence in *S*. *flexneri*, *H*. *influenzae*, *H*. *parasuis*, and *B*. *pseudomallei* [6,14–16]. Thus, the effect of the *mlaA* knockout on bacterial virulence differs between bacterial species. A previous study reported that in *N*. *gonorrhoeae*, knockout of *mlaA* sensitizes bacteria to antimicrobial peptides (defensin and polymyxin), vancomycin, and ampicillin, but increases OMV production; the authors speculated that the increased OMV could be advantageous for bacteria to survive in the mouse lower genital tract [7]. In *V*. *cholerae*, knockout of *mlaA* increases OMV production and alters the lipid composition of the outer membrane, which could be beneficial for bacteria adaptation in host environment containing antimicrobial peptides and bile acids [18]. In *P*. *aeruginosa*, knockout of *mlaA* increases bacterial virulence *via* ZnuA, which functions in Zn incorporation [8]. The effect of the *mlaA* knockout on OMV production was not examined in *P*. *aeruginosa*. Thus, the increased OMV production in the *mlaA*-knockout mutant is conserved among *E*. *coli*, *N*. *gonorrhoeae*, and *V*. *cholerae*, and could underlie the increased virulence of the *mlaA-*knockout mutant. It should also be noted that the animal infection models were different among the studies of different bacterial species, and the host environment could be differently involved in the bacterial virulence phenotypes of these *mlaA-*knockout bacteria.

The *mlaA*-knockout mutant of *E*. *coli* exhibited sensitivity to many antimicrobial molecules, including levofloxacin, chloramphenicol, oxacillin, CTAB, and cholic acid, but was resistant to vancomycin (**Fig 2**), chlorhexidine [12], and arenicin-3 [13]. Because the conserved chemical structure or conserved target molecules between vancomycin, chlorhexidine, and arenicin-3 is not known, it is difficult to understand the mechanism by which the *mlaA*-knockout mutant exhibits resistance to these 3 antimicrobial molecules, while it exhibits sensitivity to other antimicrobial molecules. We speculate that there are 3 possible reasons, as follows: (i) Because OMV have the capacity to absorb various antimicrobial molecules, the increased OMV production in the *mlaA*-knockout mutant may contribute to the resistance to these antimicrobial molecules. (ii) The *mlaA* knockout increases the amount of phospholipids in the outer leaflet of the outer membrane, which could alter the permeability of antimicrobial molecules and affect bacterial sensitivity to antimicrobial molecules. (iii) MlaA may transport phospholipids bound to antimicrobial molecules from the outer leaflet of the outer membrane to the inner membrane. The knockout of *mlaA* may block the transport of antimicrobial molecules from the outer membrane to the inner membrane. These possibilities should be addressed in future studies.

By constructing multiple gene deletion mutants of *pldA*, *pagP*, and *mlaA*, which respectively maintains the lipid asymmetry of the outer membrane, we revealed that the *mlaA*/*pldA* double knockout mutant, compared with the *mlaA*-knockout mutant, had increased resistance to vancomycin. We also revealed that, compared with the *mlaA*-knockout mutant, the *mlaA*/*pldA* double knockout mutant did not have increased killing activity in silkworms. Thus, *pldA* has a role with *mlaA* in vancomycin resistance, but no role in the silkworm-killing activity. Our analysis suggests the *mlaA* is the main factor among *pldA*, *pagP*, and *mlaA* that affects the vancomycin resistance and virulence of *E*. *coli*.

MlaA is attracting attention as a drug target, because *mlaA* knockout increases the sensitivity of many bacteria to various antibiotics. The present study, however, demonstrated that the *mlaA* knockout confers *E*. *coli* resistance to vancomycin and exhibits high virulence in silkworms. Investigating the molecular mechanisms of how the *mlaA* knockout upregulates bacterial virulence will help to elucidate the biological significance of the Mla system, and contribute to the evaluation of MlaA as a drug target.

## Materials and methods

### Bacteria and culture condition

*E*. *coli* KP7600 strain and its gene-knockout mutants were cultured on LB agar medium and the bacterial colonies were aerobically cultured in LB liquid medium at 37˚C. *E*. *coli* strains transformed with pCP20 or pMW118 were cultured on LB agar plates containing 100 μg/ml ampicillin. The details of the bacterial strains and plasmids used in this study are provided in Table 2.

**Table 2:** List of bacterial strains and plasmids used.

Strain or plasmid	Genotypes or characteristics	Source or reference
Strains		
KP7600	W3110 type-A, F^-^, *lacI*^q^, *lacZ*ΔM15, λ^-^, *galK2*, *galT22*, IN(*rrnD-rrnE*)*1*	NBRP
JD strains	Transposon library using mini-Tn*10*; Kan^r^	NBRP
JD23323	KP7600 *mlaA*::mini-Tn*10*; Kan^r^	NBRP
JW3794	BW25113 Δ*pldA*::*kan*; Kan^r^	NBRP
JW0617	BW25113 Δ*pagP*::*kan*; Kan^r^	NBRP
JW2343	BW25113Δ*mlaA*:: *kan*; Kan^r^	NBRP
N0001	KP7600 Δ*pldA*:: *kan*; Kan^r^ (transduced from Keio collection JW3794)	This study
N0002	KP7600 Δ*pagP*:: *kan*; Kan^r^ (transduced from Keio collection JW0617)	This study
N0003	KP7600 *mlaA*::mini-Tn*10*; Kan^r^, Δ*pldA*::markerless	This study
N0004	KP7600 *mlaA*::mini-Tn*10*; Kan^r^, Δ*pagP*::markerless	This study
N0005	KP7600 *mlaA*::mini-Tn*10*; Kan^r^, Δ*pldA*::markerless, Δ*pagP*::markerless	This study
N0006	KP7600 Δ*mlaA*:: *kan*; Kan^r^ (transduced from Keio collection JW2343)	This study
N0006	KP7600 *mlaA*::markerless	This study
Plasmids		
pMW118	A low copy plasmid, Amp^r^	Nippongene
pMW218	A low copy plasmid, Kan^r^	Nippongene
pMW118-mlaA	pMW118 with *mlaA*, Amp^r^	This study
pMW218-mlaA	pMW218 with *mlaA*, Kan^r^	This study
pCP20	FLP recombinase, Amp^r^	[26]

Kan: Kanamycin, Cm: Chloramphenicol, Amp: Ampicillin.

### Silkworm infection experiment

Third instar silkworms (Fu/Yo X Tsukuba/Ne) were purchased from Ehime Sansyu (Ehime, Japan). The silkworms were fed an artificial diet (Silkmate, Nosan, Japan) and maintained at 27˚C. Fifth instar silkworms were fed an antibiotic-free artificial diet (Sysmex) for 1 day and used for the infection experiment. *E*. *coli* overnight culture was centrifuged at 4050 *g* for 10 min, and the precipitated cells were suspended in 0.9% NaCl. Silkworms were injected with the bacterial solution using 1-ml syringes equipped with a 27-gauge needle *via* the intra-hemolymph route [27] and maintained at 37˚C. Silkworm survival was measured every ~12 h after the injection. The OD_600_ values of the bacterial solutions were measured before the injection to confirm that the number of bacteria did not differ between samples. The number of bacteria was determined by plating bacterial solution on LB agar plates.

### Genetic manipulation

To construct gene-knockout mutants of *mlaA*, *pldA*, and *pagP*, transduction using a phage P1 *vir* was performed. First, the *pldA* knockout strain (N0001) or *pagP* knockout strain (N0002) were constructed by transduction from JW3794 or JW0617 to KP7600 strain. After the transduction step, the kanamycin resistance marker was removed by transformation with pCP20 expressing FLP recombinase, and pCP20 was removed by culturing the bacteria at 43˚C. Next, the *pldA/mlaA* double knockout strain (N0003) or *pagP/mlaA* double knockout strain (N0004) were constructed by transduction from JD23323 to the *pldA* or *pagP* markerless knockout strains. Third, the *pldA/pagP* double knockout strain was constructed by transduction from JW0617 to the *pldA* markerless knockout strain. The kanamycin resistance marker was removed from the *pldA/pagP* double knockout strain by transformation with pCP20, and the transduction was performed from JD23323 to the markerless *pldA/pagP* double knockout strain, resulting in the *mlaA/pldA/pagP* triple knockout strain. The gene knockouts in the mutant strains were confirmed by PCR. To construct a plasmid carrying the *mlaA* gene, the DNA fragment encoding the *mlaA* gene was amplified by PCR using primer pairs (forward, 5’-TCTTCTAGACCGCAGTCACGGTATTTTC-3’, reverse, 5’-GGTGGTACCTGGTTCCGATCATCAGGTT-3’) from genomic DNA of KP7600 as a template. The amplified DNA fragment was cloned into XbaI and KpnI sites of pMW118 or pMW218, resulting in pMW118-mlaA or pMW218-mlaA.

### Evaluation of bacterial resistance to antimicrobial substances

To measure bacterial resistance to antibiotics and detergents, autoclaved LB agar medium was mixed with antibiotics or detergents and poured into square plastic dishes (Eiken Chemical, Tokyo, Japan). *E*. *coli* overnight cultures were serially diluted 10-fold in a 96-well microplate and 5 μl of the diluted bacterial solution was spotted onto the LB agar plates supplemented with drugs. The plates were incubated at 37˚C for 1 day and colonies were photographed using a digital camera.

Bacterial resistance to silkworm hemolymph was measured according to our previous method [24]. Briefly, fifth instar silkworms were injected with heat-killed *E*. *coli* KP7600 cells and the hemolymph was collected at 1 day after inoculation. The collected hemolymph was frozen in liquid nitrogen and stored at -80˚C. Overnight cultures (10 μl) of *E*. *coli* strains (Parent/pMW118, *mlaA*::Tn/pMW118, or *mlaA*::Tn/pMW118-mlaA) were inoculated into fresh LB medium (1 ml) and aerobically cultured at 37˚C. The silkworm hemolymph (27 μl) was added to the culture at 40 min after the inoculation and the OD_600_ was measured every 1 h.

### Preparation of OMV

*E*. *coli* overnight culture (1 ml) was inoculated into 100 ml of LB medium in a flask and aerobically cultured at 37 ˚C for 24 h. The culture was centrifuged at 4450 *g* for 10 min, and the supernatant was filtered through a 0.22-μm polyvinylidene difluoride membrane (Millipore). The supernatant was centrifuged at 45,000 *g* for 3 h and the precipitate was dissolved with SDS sample buffer. The sample was electrophoresed in 15% SDS polyacrylamide gel and the gel was stained with Coomassie brilliant blue.

### Western blot analysis

The OMV samples were electrophoresed in 15% SDS polyacrylamide gel, and blotted to a PVDF membrane (Immobilon-P, Millipore). The membrane was treated with TBST (20 mM Tris-HCl [pH7.6], 150 mM NaCl, 0.12% Tween20) containing 5% skim milk for 1 h. The membrane was treated with a TBST buffer containing 1:5000 anti-OmpA IgG (111120, Antibody research corp., MO, USA) or 1:10000 anti-LPS core (WN1 222–5, Hycult Biotech, Uden, The Netherlands) for 1 h at room temperature. After washing with TBST, the membrane was treated with TBST containing 1:5000 anti-rabbit IgG conjugated with horseradish peroxidase HRP or 1:5000 anti-mouse IgG conjugated with HRP for 1 h at room temperature. After washing with TBST, the membrane was treated with a HRP substrate (Western Lightning Plus-ECL, Perkin Elmer). The signal was visualized using ImageQuant LAS 4000 (Fujifilm, Tokyo, Japan). The band intensity was measured by Image J software [28].

### Statistical analysis

Differences of the growth curves in the presence of silkworm hemolymph were assessed using the Student *t* test in Microsoft Excel for Mac (version 16.56). Statistical analyses of the survival curves of silkworms were performed using the log rank test with GraphPad PRISM software (version 5.0c).

## Supporting information

S1 Raw images Original uncropped images for SDS polyacrylamide gel electrophoresis and western blots. (PDF)
