# Supplementary figures and images for: Knockout of mlaA increases Escherichia coli virulence in a silkworm infection model
Source: PLoS One. 2022 Jul 13;17(7):e0270166. doi: 10.1371/journal.pone.0270166 (PMC9278758; doi:10.1371/journal.pone.0270166)

Raw\_image for Fig 3A

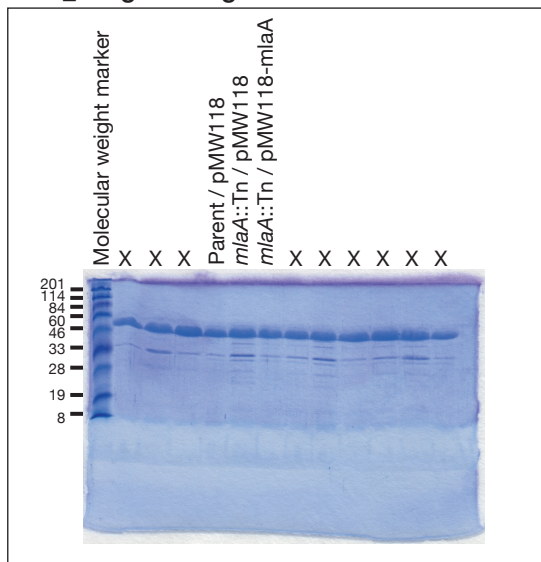

Raw\_image for Fig 3B

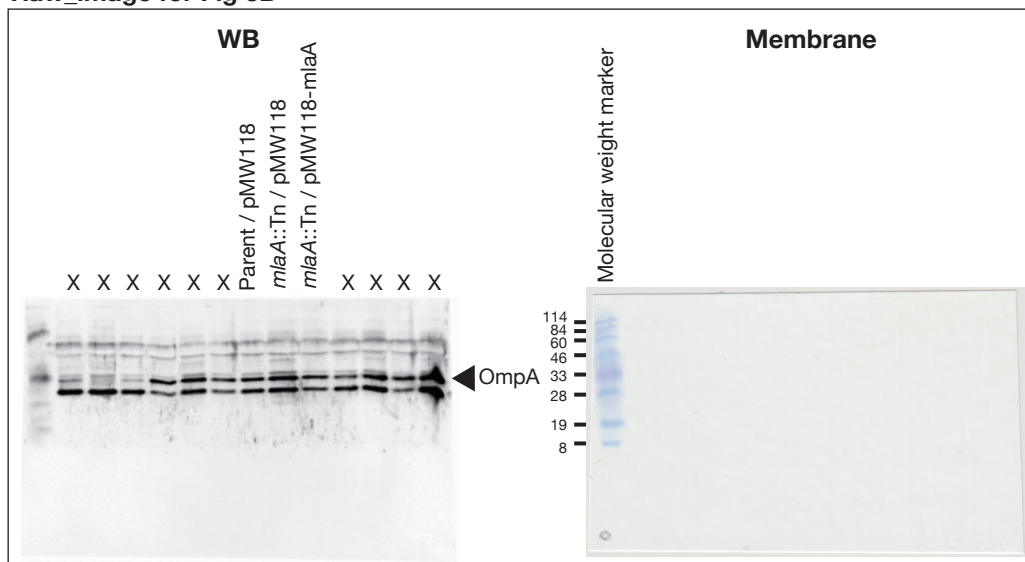

Raw\_image for Fig 3C

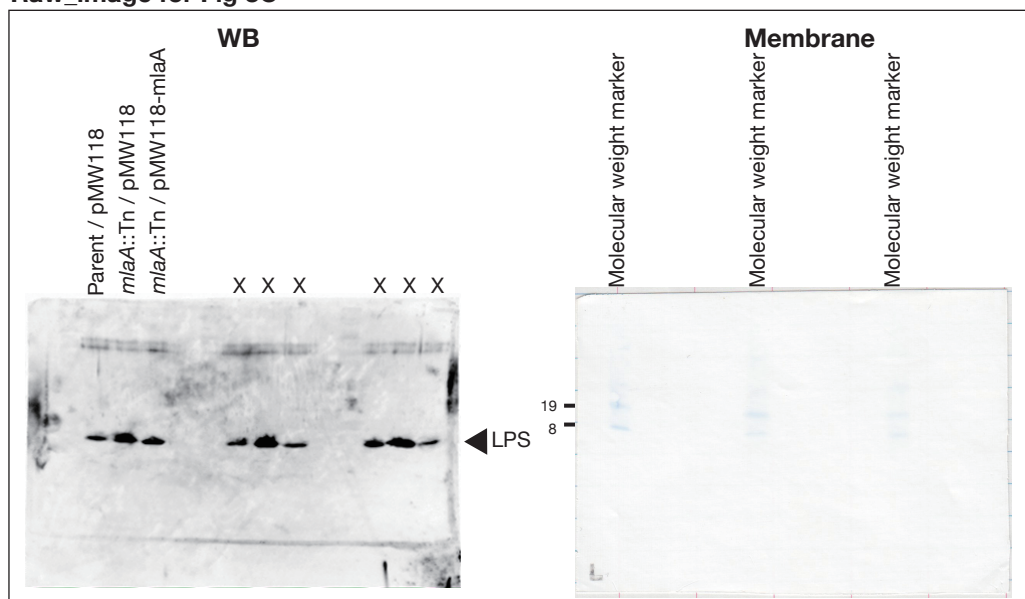

Supplement: S1 Raw images — (PDF) [file pone.0270166.s001.pdf]
